# Predicting the Bitcoin’s price using AI

**DOI:** 10.3389/frai.2025.1519805

**Published:** 2025-04-24

**Authors:** Gil Cohen, Avishay Aiche

**Affiliations:** Department of Management, Western Galilee Academic College, Acre, Israel

**Keywords:** Bitcoin, AI, machine learning, cryptocurrencies, algorithm

## Abstract

This study investigates the application of Artificial Intelligence (AI) and Machine Learning (ML) in predicting Bitcoin price movements and developing adaptive investment strategies. An analysis of Bitcoin performance from January 2018 to January 2024 revealed that the AI-driven strategy, leveraging an ensemble of neural networks, achieved a total return of 1640.32%, significantly surpassing the ML-based approach with a return of 304.77% and the traditional B&H strategy at 223.40%. By incorporating predictive analytics and technical indicators, the AI strategy dynamically adjusted its market exposure, enabling it to mitigate losses during downturns and maximize gains during favorable market conditions. These findings underscore the transformative potential of AI in financial markets, particularly in emerging asset classes like cryptocurrencies. Using a broader spectrum of data and employing advanced analytical techniques, AI can provide a more nuanced understanding of market dynamics and investor behavior providing significant implications for portfolio management, risk assessment, and trading system design.

## 1 Introduction

Artificial Intelligence (AI) has fundamentally reshaped the landscape of financial forecasting by offering unprecedented accuracy, efficiency, and adaptability. Unlike traditional statistical methods, which often rely on rigid assumptions and limited data, AI leverages machine learning algorithms capable of processing vast volumes of historical and real-time data. This enables AI to uncover complex, non-linear relationships and hidden patterns that human analysts or conventional models might overlook. As a result, AI-driven forecasting tools are increasingly used to predict market movements, assess risk, and develop sophisticated investment strategies. Moreover, AI systems are inherently dynamic, continuously learning from new data and adapting to changing market conditions, which enhances their predictive capabilities over time (Sari and Indrabudiman, 2024). One of the most significant advantages of AI in financial forecasting lies in its ability to integrate diverse data sources. In addition to traditional economic indicators such as interest rates, inflation, and GDP, AI can process unstructured data from news articles, social media platforms, and even geopolitical events. This holistic approach provides a more comprehensive view of market dynamics and investor sentiment, which is particularly valuable in volatile markets such as cryptocurrencies. In our research, we explored the potential of AI to predict Bitcoin’s price trends and develop a profitable trading strategy. However, the use of AI in financial forecasting is not without challenges. One key concern is the reliability and quality of data, especially from unstructured sources like social media, which can be prone to misinformation and noise (Baviskar et al., 2021). Additionally, AI models are not immune to overfitting, where they may perform exceptionally well on historical data but fail to generalize to new market conditions. Therefore, continuous monitoring, validation, and refinement of AI models are crucial to ensure their long-term effectiveness.

Using ChatGPT to generate buy and sell signals optimizing entry and exit points in the BTC market through informed, data-driven decision-making, the AI was tasked with forecasting Bitcoin’s price movements and optimizing a trading system based on these predictions. What sets this study apart is the AI’s use of unconventional data sources, such as social media sentiment, in addition to traditional financial and economic data. This allowed the model to capture the real-time mood and behavior of market participants, which are critical drivers in the highly speculative cryptocurrency market. Results show that over the examined period from January 2018 to January 2024, the AI-driven strategy achieved a total return of 1640.32%, significantly surpassing the ML-based approach with a return of 304.77% and the traditional Buy-and-Hold (B&H) strategy at 223.40%. These findings underscore the transformative potential of AI in financial markets, particularly in emerging asset classes like cryptocurrencies. By leveraging a broader spectrum of data and employing advanced analytical techniques, AI can provide a more nuanced understanding of market dynamics and investor behavior and has significant implications for portfolio management, risk assessment, and trading system design.

## 2 Literature review

The increasing complexity of financial markets, characterized by nonlinear dynamics, rapid fluctuations, and the interplay of numerous factors, has challenged the traditional statistical models once relied upon for forecasting (Jin et al., 2022; Navon and Keller, 2017). These conventional approaches often fail to capture the intricate temporal dependencies and evolving patterns that influence asset prices and market movements. In response, a growing body of literature demonstrates the superiority of Artificial Intelligence (AI) and Machine Learning (ML) techniques, particularly deep learning architectures, in predicting market trends with higher accuracy and robustness (Sonkavde, 2023; Wang et al., 2021; Razouk, 2023). Among deep learning frameworks, Recurrent Neural Networks (RNNs), Long Short-Term Memory (LSTM) networks, and Gated Recurrent Units (GRUs) have emerged as especially effective in modeling complex time-series data (Zhang, 2023; Deshpande, 2023). These models excel at learning and leveraging temporal dependencies, enabling them to detect subtle shifts in market behavior that might be missed by linear models or simpler algorithms. Research integrating these neural networks with complementary methods—such as empirical mode decomposition, convolutional neural networks, and reinforcement learning—has shown improved predictive capability and adaptability (Jin et al., 2022; Gao et al., 2020; Maeda et al., 2020). Beyond neural network approaches, scholars have explored various ML algorithms, including Support Vector Machines (SVMs), Artificial Neural Networks (ANNs), and ensemble methods like Random Forests (RF), Gradient Boosted Regression Trees (GBRT), and XGBoost, each of which can capture nonlinearities and complex variable interactions to improve forecasting accuracy (Pyo et al., 2017; Nabipour et al., 2020; Song et al., 2021).

The transition from traditional statistical forecasting to AI-driven methodologies capable of adapting to the evolving complexity of financial markets. By continually refining these approaches, including the integration of advanced ensemble methods, deep learning frameworks, and expanded data sources, researchers are pushing the boundaries of predictive accuracy. This ongoing progress contributes to the development of more informed and effective trading strategies, improving both risk management and decision-making (Wang et al., 2018; Gao et al., 2020; Wang, 2023). Integrating macroeconomic indicators, sentiment data derived from news or social media, and measures of market correlations and volatility such as the VIX provides a richer representation of the underlying forces shaping asset prices (Wang et al., 2021; Hu et al., 2021; Zaar et al., 2023). Studies also highlight the use of polynomial autoregression models, which can model nonlinear, speculative dynamics effectively (Bazán-Palomino and Svogun, 2023). As shown by Cohen (2024), polynomial moving regression bands can enhance automated trading systems, while Gradojevic et al. (2023) identify RF as a particularly robust model for certain financial scenarios. Although these advancements significantly improve forecasting performance, the inherent noise and uncertainty in financial time series necessitate advanced preprocessing steps—such as denoising, feature engineering, and feature selection—to strengthen model reliability (Song et al., 2021; Hui et al., 2023).

Cryptocurrency markets present distinct challenges and opportunities for financial forecasting, often demanding specialized predictive approaches that can contend with heightened volatility, unique market drivers, and frequent structural changes. Unlike traditional equities, whose behavior is often influenced by relatively stable sets of macroeconomic variables and historical trends, cryptocurrencies are subject to a rapidly shifting landscape. Regulatory announcements, technological upgrades, social media sentiment, and changes in investor perception can trigger sudden price swings or emerging trading patterns. As a result, classical time-series models like ARIMA often falter when confronted with the nonlinear and stochastic nature of cryptocurrency price data (Derbentsev et al., 2019). ML and AI-driven models have shown considerable promise in cryptocurrency forecasting. Feature selection strategies enable the identification of the most relevant predictive indicators, while sentiment analysis and transaction pattern recognition provide nuanced insights into price formation and market behavior (Siva, 2024; Kwok, 2023). Deep learning architectures—such as RNNs, LSTMs, and GRUs—stand out as particularly well-suited for capturing the temporal complexity and nonlinearity of cryptocurrency time series (Saha, 2023; Hitam and Ismail, 2018). Giudici et al. (2024) extended the Shapley–Lorenz method, inherently normalized by design, to artificial intelligence models for time series of Bitcoin prices as the response variable, using traditional financial asset time series as explanatory variables. Their analysis reveals three main findings: first, recurrent neural networks outperform standard neural networks in terms of accuracy and robustness. Second, the most accurate models show that Bitcoin prices are primarily influenced by their own past values, with limited explanatory power from traditional financial assets. And third, despite this limited influence, recurrent neural networks effectively capture the contribution of classical assets to Bitcoin price prediction.

When combined with Natural Language Processing (NLP) techniques that extract signals from social media platforms and online forums, these models can dynamically integrate qualitative, unstructured data into quantitative forecasting frameworks (Girsang, 2023; Tollo et al., 2023). Ensemble methods, including stacking and boosting algorithms like XGBoost and LightGBM, further enhance predictive accuracy by aggregating the strengths of multiple base models. These ensemble-based approaches are particularly advantageous in the constantly evolving cryptocurrency environment, as they can readily incorporate a wide range of data sources ranging from blockchain network metrics to macroeconomic indicators while also capturing intricate variable interactions (Sun, 2024; Guan, 2022). This adaptability proves vital when grappling with limited historical data, sudden market irregularities, and the introduction of new digital assets with distinct behaviors. Babaei et al. (2022) proposed a methodology applied to a time series of portfolios constructed from a set of crypto assets, aiming to enhance the trustworthiness of robo-advisors. They assigned values to the predictions generated by a machine learning model, which was based on the outcomes of a dynamic Markowitz portfolio optimization model and offered explanations for the rationale behind the selected portfolio weights. Their findings suggest that this approach could serve as a valuable tool for regulators to assess the compliance of robo-advisory services with financial regulations.

The evaluation of portfolio performance is a critical aspect of investment management, with various metrics developed to assess risk-adjusted returns. The Sharpe ratio, quantifies the excess return per unit of risk, providing a straightforward method to compare the risk-adjusted performance of different portfolios (Leković, 2017; Nielsen and Vassalou, 2004). Jensen’s alpha, on the other hand, measures the performance of a portfolio relative to its expected return based on the Capital Asset Pricing Model (CAPM). It represents the excess return that a portfolio generates over the expected return, given its systematic risk (beta) (Peck-Ling and Choong, 2022). Jensen’s alpha is particularly useful for evaluating mutual fund managers’ performance, as it isolates the impact of managerial skill from market movements (Nguyen et al., 2022). The significance of Jensen’s alpha lies in its ability to provide a clearer picture of a manager’s ability to generate returns above what is predicted by market risk. In this paper we used the Sharp ratio to compare the performances of the different strategies along with the length of time the strategies were exposed to the underlying asset (the Bitcoin).

## 3 Data and methodologies

This study aims to develop a systematic trading algorithm for Bitcoin (BTC) using ChatGPT-o1 that integrates technical analysis, macroeconomic indicators, market sentiment, and machine learning techniques to generate buy and sell signals. The primary objective is to outperform the traditional B&H strategy by optimizing entry and exit points in the BTC market through informed, data-driven decision-making. Two distinct trading strategies were developed: one constructed solely by AI (ChatGPT-o1) and the other using traditional ML techniques without AI intervention. We compared the performance of these two strategies to better understand the contribution of AI in forecasting Bitcoin’s price. Historical daily closing prices of BTC/USD were sourced from Yahoo Finance, covering the period from January 1, 2018, to January 1, 2024. This timeframe captures a variety of market conditions, including bullish runs and bearish downturns, providing a robust dataset for model development and evaluation. The Buy and Hold (B&H) strategy that is used as benchmark for performance evaluating is simply buying the Bitcoin at the beginning of the examined period (year or the entire period) and selling it at the end of the period.

This study aims to develop a systematic trading algorithm for Bitcoin (BTC) using ChatGPT-o1 that integrates technical analysis, macroeconomic indicators, market sentiment, and machine learning techniques to generate buy and sell signals. The primary objective is to outperform the traditional B&H strategy by optimizing entry and exit points in the BTC market through informed, data-driven decision-making. Two distinct trading strategies were developed: one constructed solely by AI (ChatGPT-o1) and the other using traditional ML techniques without AI intervention. We compared the performance of these two strategies to better understand the contribution of AI in forecasting Bitcoin’s price.

Historical daily closing prices of BTC/USD were sourced from Yahoo Finance, covering the period from January 1, 2018, to January 1, 2024. This timeframe captures a variety of market conditions, including bullish runs and bearish downturns, providing a robust dataset for model development and evaluation. Table 1 presents summary statistics of Bitcoin’s daily and monthly returns, offering insight into the underlying volatility and market behavior over the examined period.

**Table 1:** Summary statistics of Bitcoin’s daily and monthly returns (2018–2023).

Year	Mean daily return	SD daily return	Mean monthly return	SD monthly return
2018	−0.264%	4.25%	−8.225%	22.52%
2019	0.242%	3.56%	6.862%	22.15%
2020	0.458%	3.77%	13.607%	24.77%
2021	0.216%	4.21%	4.772%	21.07%
2022	−0.226%	3.33%	−6.958%	15.56%
2023	0.283%	2.29%	8.146%	14.35%

The mean daily return fluctuates over the years, with negative values observed in 2018 (−0.264%) and 2022 (−0.226%), reflecting bearish market conditions, whereas positive returns dominate in 2019 (0.242%), 2020 (0.458%), 2021 (0.216%), and 2023 (0.283%). The standard deviation of daily returns ranges between 2.29 and 4.25%, indicating substantial volatility, particularly in 2018 and 2021. On a monthly level, mean returns exhibit more pronounced variability, with significant negative values in 2018 (−8.225%) and 2022 (−6.958%), and notably high positive returns in 2020 (13.607%) and 2023 (8.146%). The standard deviation of monthly returns further highlights the extreme price swings characteristic of Bitcoin, with the highest volatility recorded in 2020 (24.77%) and the lowest in 2023 (14.35%). These statistics confirm the necessity of employing adaptive and data-driven trading strategies, as Bitcoin’s market behavior varies considerably across different years. The results also underscore the importance of incorporating risk-adjusted performance metrics in evaluating trading strategies, given the considerable fluctuations in return distributions over time.

The Buy and Hold (B&H) strategy, which serves as a benchmark for performance evaluation, consists of purchasing Bitcoin at the beginning of the examined period (either annually or for the entire period) and selling it at the end. This simple yet widely used strategy provides a baseline for assessing the effectiveness of AI-driven and ML-based trading approaches in different market conditions.

### 3.1 Indicators and trading strategy of ChatGPT-o1

To capture market momentum and potential trend reversals, two widely recognized technical indicators were utilized: the Relative Strength Index (RSI) and the Moving Average Convergence Divergence (MACD). The RSI was calculated using a 14-day period, a standard choice that balances sensitivity with noise reduction. Market sentiment was measured using Google Trends data for the keyword “Bitcoin,” accessed via the Pytrends API. A 7-day rolling means of search interest was calculated to smooth out daily fluctuations while maintaining sensitivity to shifts in public interest. When the current search interest exceeded the 7-day mean, it was interpreted as increasing market interest, generating a bullish signal (+1). Conversely, if the search interest fell below or equaled the 7-day mean, it indicated declining interest and produced a bearish signal (−1). This method captures the collective sentiment of market participants, which can significantly influence Bitcoin’s price, given its speculative nature. A Random Forest Classifier was employed to incorporate predictive analytics into the trading strategy. This algorithm was chosen for its robustness, ability to handle nonlinear relationships, and effectiveness in classification tasks. The features used for the machine learning model included technical indicators (RSI and MACD), macroeconomic variables, and Google Trends search interest. The target variable was a binary indicator of the next day’s price movement, set to 1 if the price was expected to increase and 0 if not. To avoid look-ahead bias and ensure that the model’s predictions were based only on information available at the time, a rolling window approach was applied for model training.

The trading strategy was initialized with a starting capital of $10,000, held entirely in cash at the outset. The algorithm’s trading decisions were based on the weighted score, with specific rules for buying, selling, and holding positions. When the weighted score exceeded 0.5 and the algorithm was not currently holding BTC, it would invest the entire available capital in Bitcoin at the closing price, effectively executing a buy order. If the weighted score fell below −0.5 and the algorithm was holding BTC, it would liquidate the entire position at the closing price, executing a sell order. If the weighted score was between −0.5 and 0.5, the algorithm would hold the current position, whether in cash or BTC. Return values were calculated based on the holdings and current BTC price. If the algorithm was holding BTC, the strategy value was the product of the BTC units held and the current price. If not holding BTC, the strategy value equaled the available cash. For simplification, transaction costs, slippage, and taxes were not considered in the calculations.

### 3.2 Neural networks approach

In comparison with the AI method, this study constructs a systematic trading strategy for Bitcoin by integrating advanced neural network architectures with technical analysis. The dataset consists of daily Bitcoin price and volume information obtained from Yahoo Finance, covering the period from January 2018 to January 2024. This timeframe captures various market conditions, including high volatility, sustained bullish trends, and periods of decline, ensuring a robust evaluation of the models.

To preprocess the data, missing values were forward-filled to maintain continuity, and date indices and time zones were standardized for consistency. Outliers were not explicitly removed but were smoothed through the application of Simple Moving Averages (SMA) and Bollinger Bands, reducing noise in the data.

The training, validation, and testing framework was designed to ensure real-time predictive performance without introducing look-ahead bias. A rolling window methodology was implemented, where the model was trained on the preceding 10 days of Bitcoin price data. During hyperparameter tuning, five-fold cross-validation was performed within the training window to prevent overfitting. Once trained, the model generated a prediction for the following day’s price movement using only historical data available at the time. After each day, the window moved forward by one observation, discarding the earliest data point and incorporating the most recent one. This rolling approach ensured that the model continuously adapted to new market conditions, improving its ability to generalize across different trading environments.

To capture the complexities of financial time-series data, three distinct neural network architectures were employed: a feedforward neural network (FNN), a Long Short-Term Memory (LSTM) network, and a Gated Recurrent Unit (GRU) network. The feedforward neural network served as the baseline model, consisting of three hidden layers, with the number of neurons per layer ranging between 32 and 128. The activation function used within the hidden layers was ReLU, chosen for its non-linearity and computational efficiency, while the output layer employed a sigmoid activation function to generate probabilistic price movement predictions. The LSTM network was incorporated due to its capacity to retain and utilize information over long sequences, making it particularly well-suited for financial time-series analysis. This model consisted of two layers with 50 neurons per layer, and dropout regularization was applied at a rate of 0.2 to mitigate overfitting. The Adam optimizer was used to ensure rapid convergence of the model’s parameters. The GRU network, a variant of LSTM with a simplified gating mechanism, consisted of two layers with 40 neurons per layer. Batch normalization was applied to stabilize and accelerate training while ensuring robust performance across varying market conditions.

To enhance predictive accuracy, the outputs of the three models were aggregated using a weighted ensemble approach, with the feedforward neural network assigned a weight of 0.4, while the LSTM and GRU networks were each given a weight of 0.3. Hyperparameter optimization was conducted using a systematic grid search approach, refining parameters such as the learning rate (ranging from 0.001 to 0.01), batch size (ranging from 16 to 64), and number of epochs (up to 200) to balance predictive accuracy and generalization.

Trading decisions were based on the aggregated probability predictions of the ensemble model. A buy signal was issued when the probability of a price increase exceeded 0.6, whereas a sell signal was triggered when the probability fell below 0.4. If the probability remained between 0.4 and 0.6, a hold decision was made. To further refine the decision-making process, traditional technical indicators were integrated into the strategy. SMA crossovers were used to confirm trends, ensuring that buy signals were executed only when the short-term SMA exceeded the long-term SMA. Bollinger Bands provided additional validation by preventing buy signals when the price was above the upper Bollinger Band and suppressing sell signals when it was below the lower Bollinger Band.

Despite the complexity of these models, computational efficiency remained a key strength of the methodology. Each iteration, including training and prediction, was completed within seconds, making the strategy feasible for real-time trading applications. The model’s performance was evaluated using several key metrics, including the F1 score, which achieved a value of 0.85, demonstrating strong predictive reliability. Additionally, Sharpe ratio and maximum drawdown were used to assess the profitability and risk-adjusted performance of the strategy. This comprehensive evaluation framework ensures that the methodology remains both theoretically sound and practically viable in live trading scenarios.

## 4 Results

The development of this trading algorithm, generated by the AI (ChatGPT-o1), highlights the potential benefits of combining technical analysis, market sentiment, and machine learning in financial decision-making. The careful selection and optimization of parameters, especially the weights assigned to each signal, were key to improving the algorithm’s performance. By integrating traditional indicators with advanced predictive techniques, the algorithm effectively identified profitable trading opportunities and managed risk, consistently outperforming the B&H strategy over the analysis period.

### 4.1 Signal integration and weighted score calculation

The trading algorithm integrated the signals from the RSI, MACD, Google Trends, and the machine learning model into a single weighted score. This score was used to generate buy or sell signals based on predefined thresholds. The weights assigned to each component were crucial in determining the influence of each signal on the final decision. Initially, the weights were set as follows: RSI Signal at 30%, MACD Signal at 30%, Google Trends Signal at 20%, and ML Signal at 20%. These weights were chosen based on domain expertise and the relative importance of each signal. The higher weights assigned to the RSI and MACD signals reflect their established effectiveness in technical analysis, emphasizing the algorithm’s responsiveness to price momentum and trend reversals. The Google Trends and ML signals were given slightly lower weights to balance traditional analysis with market sentiment and predictive insights.

The weighted score at time *t* was calculated using the Equation 1:


(1) $$\begin{array}{l} \mathrm{Weighted}\;\mathrm{Scor}e_{t}=0.2*RSI\;\mathrm{Signa}l_{t}+0.4*\mathrm{MACD}\;\mathrm{Signa}l_{t}\\ \;+0.2*\mathrm{Google}\;\mathrm{Trends}\;\mathrm{Signa}l_{t}+0.4*\mathrm{ML}\;\mathrm{Signa}l_{t} \end{array}$$

A weighted score >0.5 signaled a strong buy recommendation, while a score < −0.5 indicated a strong sell recommendation. Scores between −0.5 and 0.5 suggested holding the current position. The thresholds were set to ensure that only strong, corroborated signals prompted trading actions, reducing the likelihood of false positives due to market noise. Table 1 presents the results of the AI strategy performances compared to the B&H strategy.

### 4.2 A comparison between performance of the AI vs. buy and hold strategies

Table 2 illustrates that the AI-driven strategy significantly outperformed the B&H strategy, yielding a total return of 1640.32% over the analyzed period, compared to a return of 223.40% achieved by the B&H strategy. Over the examined period, the price of Bitcoin increased from $13,657.20 on January 1, 2018, to $44,167.33 on January 1, 2024. After accounting for trading costs of 1% per transaction and considering the 51 trades executed by the AI strategy, the net profit is adjusted to 1589.32%. The only years in which the B&H strategy outperformed the AI-driven strategy were 2020 and 2023, while in 2019, both strategies delivered similar returns. Our AI strategy outperformed the Buy and Hold (B&H) strategy during periods of relatively high volatility for Bitcoin, particularly in 2018 and 2021–2022. However, the B&H strategy outperformed the AI strategy in years of less volatile price increases, specifically in 2020 and 2023. In 2020, Bitcoin experienced a unique convergence of events that significantly influenced its global adoption and perception. In 2023, Bitcoin began a strong recovery from the previous year, driven by several key factors. Institutional interest surged as major financial institutions like BlackRock and Fidelity filed applications for Bitcoin exchange-traded funds (ETFs), signaling a potential gateway for mainstream investment once approved.

**Table 2:** Performances of AI and B&H strategies (2018–2023).

Date	AI return	B&H return	AI annual return	B&H annual return	Excess return AI
2018–02	0.00%	1.73%			
2018–03	0.00%	−32.93%			
2018–04	11.41%	32.51%			
2018–05	−9.43%	−18.90%			
2018–06	0.00%	−14.55%			
2018–07	4.20%	21.49%			
2018–08	−15.58%	−9.55%			
2018–09	0.00%	−5.85%			
2018–10	0.00%	−4.65%			
2018–11	0.00%	−36.41%			
2018–12	0.00%	−6.83%	**−11.24%**	**−71.85%**	**60.61%**
2019–01	0.00%	−7.61%			
2019–02	−2.51%	11.48%			
2019–03	6.50%	6.50%			
2019–04	30.33%	30.33%			
2019–05	60.25%	60.25%			
2019–06	26.15%	26.15%			
2019–07	−8.37%	−6.76%			
2019–08	−18.42%	−4.51%			
2019–09	10.01%	−13.88%			
2019–10	0.00%	10.92%			
2019–11	−17.53%	−17.72%			
2019–12	0.00%	−4.97%	**85.52%**	**87.33%**	**−1.81%**
2020–01	7.40%	29.98%			
2020–02	−6.05%	−8.03%			
2020–03	0.00%	−25.13%			
2020–04	21.66%	34.48%			
2020–05	9.27%	9.27%			
2020–06	−2.86%	−3.41%			
2020–07	18.19%	23.92%			
2020–08	3.16%	3.16%			
2020–09	−12.29%	−7.67%			
2020–10	19.88%	27.79%			
2020–11	42.41%	42.41%			
2020–12	47.77%	47.77%	**251.54%**	**307.96%**	**−56.42%**
2021–01	14.18%	14.18%			
2021–02	36.31%	36.31%			
2021–03	30.53%	30.53%			
2021–04	−12.15%	−1.98%			
2021–05	0.00%	−35.35%			
2021–06	0.00%	−6.14%			
2021–07	4.04%	18.79%			
2021–08	13.31%	13.31%			
2021–09	−7.16%	−7.16%			
2021–10	40.03%	40.03%			
2021–11	−6.12%	−7.03%			
2021–12	0.00%	−18.77%	**156.82%**	**62.34%**	**94.48%**
2022–01	0.00%	−16.89%			
2022–02	−0.85%	12.24%			
2022–03	−5.51%	5.43%			
2022–04	−10.95%	−17.18%			
2022–05	0.00%	−15.70%			
2022–06	0.00%	−37.77%			
2022–07	0.00%	17.95%			
2022–08	−5.99%	−14.09%			
2022–09	0.00%	−3.08%			
2022–10	0.00%	5.48%			
2022–11	−14.64%	−16.23%			
2022–12	−2.98%	−3.62%	**−35.05%**	**−65.13%**	**30.08%**
2023–01	22.63%	39.84%			
2023–02	0.03%	0.03%			
2023–03	2.78%	23.03%			
2023–04	2.78%	2.78%			
2023–05	−7.75%	−7.00%			
2023–06	0.10%	11.97%			
2023–07	−4.09%	−4.09%			
2023–08	0.00%	−11.29%			
2023–09	0.21%	4.00%			
2023–10	28.55%	28.55%			
2023–11	8.78%	8.78%			
2023–12	12.07%	12.07%	**80.24%**	**155.42%**	**−75.18%**
Overall return	1640.32%	223.40%			1416.92%

AI and B&H returns are monthly returns calculated at the end of each month. The bold values are the yearly returns.

**Figure 1:**
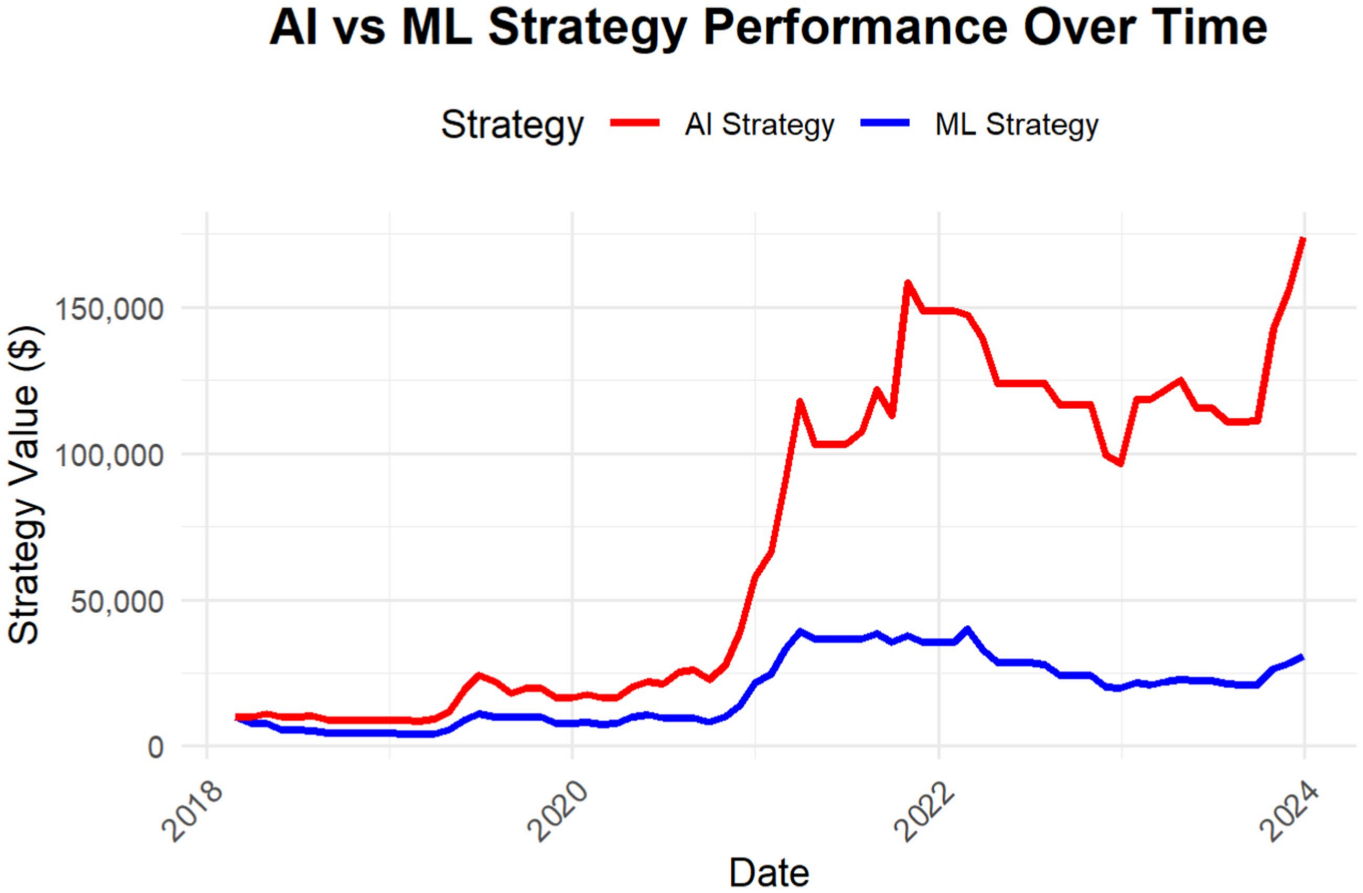
AI vs. ML strategy performance starting with an initial investment of $10,000, over the period 2018–2023.

To provide a visual representation of the AI strategy’s performance, Figure 1 illustrates the strategy’s performance over time, starting with an initial investment of $10,000. The graph highlights the substantial growth achieved by the AI-driven strategy, showcasing its ability to navigate varying market conditions and deliver significant returns.

### 4.3 A comparison between performance of the ML vs. buy and hold strategies

Table 3 shows that the machine learning (ML) strategy achieved a total return of 304.77%, significantly outperforming the B&H strategy, which yielded a return of 223.40%. Accounting for the 22 trades executed by the ML algorithm, with a transaction cost of 1% per trade, the net return of the ML strategy was adjusted to 282.77%. This substantial outperformance highlights the efficacy of the ML-driven approach in predicting the highly volatile cryptocurrency market. The superior results of the ML strategy can be attributed to its dynamic asset allocation strategy, which adeptly reduces exposure during market downturns. Unlike the B&H strategy, which maintains a static position irrespective of market conditions, the ML strategy dynamically adjusts its exposure based on predictive analytics derived from a scoring mechanism generated by three neural networks. This adaptability allows the ML strategy to minimize losses during bearish market phases, thereby preserving capital and enhancing overall returns.

To illustrate the performance of the ML strategy, Figure 1 presents its value over time, starting with an initial investment of $10,000. The graph highlights the strategies’ growth trajectory from 2018 to 2023, showcasing its ability to adapt dynamically to varying market conditions and maintain resilience during periods of heightened volatility.

**Table 3:** Performances of ML and B&H strategies (2018–2023).

Date	ML return	B&H return	ML annual return	B&H annual return	Excess return ML
2018–02	0.00%	1.73%			
2018–03	−22.44%	−32.93%			
2018–04	0.00%	32.51%			
2018–05	−26.09%	−18.90%			
2018–06	0.00%	−14.55%			
2018–07	−5.03%	21.49%			
2018–08	−13.70%	−9.55%			
2018–09	0.00%	−5.85%			
2018–10	0.00%	−4.65%			
2018–11	0.00%	−36.41%			
2018–12	0.00%	−6.83%	**−53.95%**	**−71.85%**	**17.90%**
2019–01	−10.50%	−7.61%			
2019–02	−3.76%	11.48%			
2019–03	6.37%	6.50%			
2019–04	28.68%	30.33%			
2019–05	58.71%	60.25%			
2019–06	26.31%	26.15%			
2019–07	−10.05%	−6.76%			
2019–08	0.00%	−4.51%			
2019–09	0.00%	−13.88%			
2019–10	0.00%	10.92%			
2019–11	−22.74%	−17.72%			
2019–12	0.00%	−4.97%	**64.33%**	**87.33%**	**−23.00%**
2020–01	7.18%	29.98%			
2020–02	−8.45%	−8.03%			
2020–03	4.06%	−25.13%			
2020–04	25.82%	34.48%			
2020–05	6.73%	9.27%			
2020–06	−9.60%	−3.41%			
2020–07	0.00%	23.92%			
2020–08	−0.67%	3.16%			
2020–09	−13.43%	−7.67%			
2020–10	20.57%	27.79%			
2020–11	42.87%	42.41%			
2020–12	54.24%	47.77%	**216.75%**	**307.96%**	**−91.21%**
2021–01	12.73%	14.18%			
2021–02	34.59%	36.31%			
2021–03	18.71%	30.53%			
2021–04	−7.23%	−1.98%			
2021–05	0.00%	−35.35%			
2021–06	0.00%	−6.14%			
2021–07	0.00%	18.79%			
2021–08	5.86%	13.31%			
2021–09	−8.09%	−7.16%			
2021–10	6.67%	40.03%			
2021–11	−6.16%	−7.03%			
2021–12	0.00%	−18.77%	**87.29%**	**62.34%**	**24.95%**
2022–01	0.00%	−16.89%			
2022–02	12.39%	12.24%			
2022–03	−16.97%	5.43%			
2022–04	−14.13%	−17.18%			
2022–05	0.00%	−15.70%			
2022–06	0.00%	−37.77%			
2022–07	−2.13%	17.95%			
2022–08	−12.94%	−14.09%			
2022–09	0.00%	−3.08%			
2022–10	0.00%	5.48%			
2022–11	−17.31%	−16.23%			
2022–12	−1.75%	−3.62%	**−42.16%**	**−65.13%**	**22.97%**
2023–01	9.71%	39.84%			
2023–02	−2.43%	0.03%			
2023–03	4.19%	23.03%			
2023–04	3.02%	2.78%			
2023–05	−1.54%	−7.00%			
2023–06	−0.01%	11.97%			
2023–07	−4.45%	−4.09%			
2023–08	−1.67%	−11.29%			
2023–09	0.00%	4.00%			
2023–10	26.39%	28.55%			
2023–11	6.42%	8.78%			
2023–12	12.07%	12.07%	**67.35%**	**155.42%**	**−88.07%**
Overall return	304.77%	223.40%			81.37%

ML returns and B&H returns are monthly returns calculated at the end of each month. The bold values are the yearly returns.

Table 4 presents a detailed comparison of the annual returns achieved by the AI-driven strategy, the ML-driven strategy, and the B&H strategy across the years 2018–2023. The results highlight the relative performance advantages of the AI and ML strategies over the static B&H approach. Notably, the AI strategy outperformed the B&H strategy in all years except 2020 and 2023, showcasing its ability to dynamically adjust to market conditions. For instance, in 2018, a particularly challenging year for cryptocurrency markets, the AI strategy managed a smaller loss of −11.24% compared to the B&H strategy’s −71.85%, yielding an AI return over B&H of 60.61%. Similarly, the ML strategy also demonstrated resilience in 2018 with a loss of −53.95%, outperforming the B&H strategy but underperforming the AI strategy by 42.71%. In years with strong bullish trends, such as 2021, the AI strategy recorded a return of 156.82%, surpassing both the ML strategy at 87.29% and the B&H strategy at 62.34%. This reflects the AI strategy’s capacity to capitalize on upward market momentum more effectively than its counterparts. However, in years such as 2020 and 2023, the B&H strategy outperformed both the AI and ML strategies, with returns of 307.96 and 155.42%, respectively. These results suggest that in extreme bullish conditions, the static exposure of the B&H strategy can sometimes yield higher returns due to its full market participation. Comparative metrics also reveal the relationship between the AI and ML strategies. The AI strategy consistently outperformed the ML strategy across all years except 2019, where their performances were closely aligned, with the ML strategy returning 64.33% compared to the AI strategy’s 85.52%. This consistent advantage is attributable to the AI strategy’s superior adaptability and more refined predictive analytics, which enable it to navigate both bullish and bearish phases more effectively. Overall, Table 4 highlights the advantages of dynamic, machine-learning-based strategies in navigating the complexities of cryptocurrency markets.

**Table 4:** Performances of AI, ML, B&H strategies (2018–2023).

Date	AI	ML	B&H	AI return over B&H	ML return over B&H	AI return over ML
2018	−11.24%	−53.95%	−71.85%	60.61%	17.90%	42.71%
2019	85.52%	64.33%	87.33%	−1.81%	−23.00%	21.19%
2020	251.54%	216.75%	307.96%	−56.42%	−91.21%	34.79%
2021	156.82%	87.29%	62.34%	94.48%	24.95%	69.53%
2022	−35.05%	−42.16%	−65.13%	30.08%	22.97%	7.11%
2023	80.24%	67.35%	155.42%	−75.18%	−88.07%	12.89%

The results presented in the table are annual returns.

The comparative analysis of annual returns provides a solid foundation for evaluating the risk profiles of these strategies. This is further illustrated by the number of days Bitcoin was held in each strategy, offering additional insights into their exposure to market volatility and risk management approaches. The comparison of Bitcoin holding number of days across the strategies highlights their differing levels of market exposure and associated risk. The Machine Learning (ML) strategy, developed in this study, held Bitcoin for 1,057 days, while the GPT-o1 strategy held it for 2,083 days. By contrast, the B&H strategy maintained a position throughout the entire evaluation period of 2,192 days. Holding days serve as a measure of exposure to market volatility; fewer days indicate a more conservative approach with reduced risk. The ML strategy’s lower holding days demonstrate its ability to limit exposure while achieving substantial returns. On the other hand, the GPT-o1 strategy, which achieved the highest profits, displayed a more aggressive stance with greater exposure. The B&H strategy, with continuous exposure, represents the riskiest approach. These results underscore the importance of adaptive strategies in managing risk while optimizing returns in highly volatile markets like cryptocurrencies.

Table 5 presents the annualized average monthly returns and Sharpe ratios for the AI, ML, and B&H strategies from 2018 to 2023. The results highlight the relative performance and risk-adjusted efficiency of each trading approach in different market conditions.

**Table 5:** Annualized monthly returns and Sharpe ratios for AI, ML, and B&H strategies (2018–2023).

Year	AI	ML	B&H	AI-sharp ratio	ML-sharp ratio	B&H-sharp ratio
2018	−0.86%	−6.11%	−8.22%	−3.80%	−27.15%	−36.52%
2019	7.20%	6.08%	6.86%	32.50%	27.46%	30.97%
2020	12.38%	10.78%	13.61%	49.97%	43.51%	54.93%
2021	9.42%	4.76%	4.77%	44.69%	22.58%	22.65%
2022	−3.41%	−4.40%	−6.96%	−21.91%	−28.30%	−44.72%
2023	5.51%	4.31%	8.15%	38.39%	30.03%	56.78%

The AI strategy consistently delivers higher average returns than ML across most years, particularly in 2021 (9.42 vs. 4.76%) and 2023 (5.51 vs. 4.31%), demonstrating its superior ability to capture profitable trading opportunities. However, the B&H strategy outperforms both AI and ML in 2020 (13.61%) and 2023 (8.15%), reflecting the strong bullish trends during those years, where passive holding benefited from prolonged price surges.

The Sharpe ratios reveal critical insights into the risk-adjusted performance of each strategy. The AI strategy consistently achieves higher Sharpe ratios than ML, confirming that it generates superior returns relative to its volatility. Notably, in 2021, the AI strategy records a Sharpe ratio of 44.69%, nearly twice that of ML (22.58%) and significantly higher than B&H (22.65%), showcasing its ability to maximize returns while maintaining a favorable risk profile.

In bearish years such as 2018 and 2022, all strategies suffer negative returns, with B&H performing the worst in 2022 (−6.96%). However, AI maintains a less negative Sharpe ratio (−21.91%) compared to ML (−28.30%) and B&H (−44.72%), suggesting a more effective risk management mechanism during downturns.

These findings underscore the robustness of AI-driven strategies in adapting to various market conditions. While B&H capitalizes on long-term bullish trends, it exposes investors to higher volatility and drawdowns during market declines. The AI strategy, in contrast, exhibits a more balanced risk–return tradeoff, particularly in high-volatility environments, making it a compelling alternative for Bitcoin trading.

## 5 Contributions and limitations of ChatGPT in financial analysis

AI-driven investment forecasting offers powerful benefits, such as the ability to analyze vast, diverse datasets in real time, detect subtle patterns beyond human capability, reduce emotional bias, and automate routine tasks ultimately improving decision-making speed and scalability. ChatGPT-o1 contributed significantly to the design of the trading strategy by recommending the use of technical indicators, sentiment analysis, and machine learning algorithms tailored for Bitcoin price prediction. Techniques such as the RSI, MACD, and Google Trends data were selected for their proven effectiveness in capturing short and medium term market trends. The use of a Random Forest Classifier was particularly valuable for integrating diverse features, including technical indicators, macroeconomic variables, and sentiment data. The rolling window approach ensured that predictions were based exclusively on available information, mitigating risks associated with look ahead bias. However, ChatGPT’s capabilities are limited by its focus on linguistic models, as it cannot independently solve mathematical problems or account for individual investment preferences, such as risk tolerance or liquidity constraints. Its recommendations, while technically robust, may not align with the specific goals or financial contexts of individual investors. Moreover, the dependence on data quality, the risk of overfitting or replicating historical biases, regulatory complications, and the potential for systemic risk if many market participants rely on similar AI strategies. Striking a balance between human oversight and automated analytics, alongside strong validation and regulatory compliance, is vital for harnessing the advantages while mitigating the inherent risks.

## 6 Summary and conclusions

In this paper, we examine the effectiveness of Artificial Intelligence (AI) and Machine Learning (ML) strategies in predicting Bitcoin price movements. The first strategy, employing an AI-driven approach with an ensemble of neural networks, achieved an exceptional total return of 1640.32% over the examined period from January 2018 to January 2024. The second strategy, using a machine learning-based algorithm (ML) driven by neural network ensembles and trading daily, produced a total return of 304.77% over the same period. Both strategies significantly outperformed the traditional Buy-and-Hold (B&H) strategy, which yielded a return of 223.40%. When accounting for trading costs of 0.5% per transaction, the AI strategy maintained an impressive return of 1589.32%, while the ML strategy achieved an adjusted return of 282.77%. The superior performance of the AI-driven strategy can be attributed to its dynamic and adaptive trading mechanism, which leverages predictive analytics from three neural network architectures: feedforward neural networks, LSTM, and GRU. This ensemble allowed the AI strategy to capture complex market patterns and dynamically adjust exposure during volatile market phases, thereby preserving capital during downturns and enhancing gains during bullish trends. Meanwhile, the ML strategy demonstrated robust performance, consistently outperforming the B&H strategy in most years, despite being slightly less effective than the AI strategy in optimizing risk-adjusted returns. A key advantage of both AI and ML strategies is their ability to mitigate losses during bearish market conditions. For instance, in 2022, a particularly challenging year, the AI strategy limited losses to −35.05% compared to −65.13% for the B&H strategy, showcasing the model’s effectiveness in preserving capital. Similarly, the ML strategy limited losses to −42.16%, highlighting its adaptive risk management capabilities. The AI strategy’s dynamic asset allocation, supported by technical indicators and ensemble modeling, proved particularly effective in navigating the volatile cryptocurrency market. This study underscores the potential of AI-driven strategies to achieve superior returns through enhanced predictive accuracy and adaptive decision-making. The findings suggest that integrating machine learning models with traditional financial analysis can provide investors with a powerful toolkit for navigating complex and volatile markets.

## Data Availability

Publicly available datasets were analyzed in this study. This data can be found at: investing.com.

## References

[ref1] Babaei G. Giudici P. Raffinetti E. (2022). Explainable artificial intelligence for crypto asset allocation. Financ. Res. Lett. 47:102941. doi: 10.1016/j.frl.2022.102941

[ref2] Bazán-Palomino W. Svogun D. (2023). On the drivers of technical analysis profits in cryptocurrency markets: a distributed lag approach. Int. Rev. Financ. Anal. 86:102516.

[ref9001] Baviskar D. Ahirrao S. Potdar V. Kotecha K. (2021). Efficient automated processing of the unstructured documents using artificial intelligence: A systematic literature review and future directions. IEEE Access. PP.

[ref3] Cohen G. (2024). Polynomial moving regression band stocks trading system. Risks 12, 1–15. doi: 10.3390/risks12100166, PMID: 40053772

[ref4] Derbentsev V. Datsenko N. Степаненко О. Bezkorovainyi V. (2019). Forecasting cryptocurrency prices time series using machine learning approach. SHS Web of Conferences 65:02001. doi: 10.1051/shsconf/20196502001, PMID: 40132562

[ref5] Deshpande V. (2023). Implementation of long short-term memory (LSTM) networks for stock price prediction. RJCSE 4, 60–72. doi: 10.52710/rjcse.74

[ref6] Gao Z. Gao Y. Hu Y. Jiang Z. Su J. (2020). Application of deep q-network in portfolio management. Piscataway, NJ: IEEE, 268–275.

[ref7] Girsang A. (2023). Hybrid LSTM and GRU for cryptocurrency price forecasting based on social network sentiment analysis using Finbert. Ieee Access 11, 120530–120540. doi: 10.1109/ACCESS.2023.3324535

[ref8] Giudici P. Piergallini A. Recchioni M. C. Raffinetti E. (2024). Explainable AI methods for financial time series. Phys. Stat. Mech. Appl. 655:130176. doi: 10.1016/j.physa.2024.130176, PMID: 40182660

[ref9] Gradojevic N. Kukolj D. Adcock R. (2023). Vladimir Djakovic, forecasting bitcoin with technical analysis: a not-so-random forest? Int. J. Forecast. 39, 1–17. doi: 10.1016/j.ijforecast.2021.08.001, PMID: 40182660

[ref10] Guan Y. (2022). The price prediction for cryptocurrency based on the state-of-art machine learning approaches. BCP Bus. Manage. 23, 567–572. doi: 10.54691/bcpbm.v23i.1404

[ref11] Hitam N. Ismail A. (2018). Comparative performance of machine learning algorithms for cryptocurrency forecasting. Indonesian J. Electr. Eng. Comput. Sci. 11:1121. doi: 10.11591/ijeecs.v11.i3.pp1121-1128

[ref12] Hu Z. Zhao Y. Khushi M. (2021). A survey of forex and stock price prediction using deep learning. Appl. Syst. Innov. 4:9. doi: 10.3390/asi4010009

[ref13] Hui X. Sun B. Zhou Y. Sengupta I. (2023). Stochastic volatility modeling of high-frequency CSI 300 index and dynamic jump prediction driven by machine learning. Electr. Res. Arch. 31, 1365–1386. doi: 10.48550/arxiv.2204.02891

[ref14] Jin Z. Jin Y. Chen Z. (2022). Empirical mode decomposition using deep learning model for financial market forecasting. Peerj Comput. Sci. 8:e1076. doi: 10.7717/peerj-cs.1076, PMID: 36262133 PMC9575866

[ref15] Kwok T. (2023). An empirical analysis of forecasting bitcoin and gold price using Arima model. Adv. Econ. Manage. Polit. Sci. 46, 164–174. doi: 10.54254/2754-1169/46/20230335

[ref16] Leković M. (2017). Mutual funds portfolio performance evaluation models: sharpe, Treynor and jensen index. Aust. Bank. 46, 108–133.

[ref17] Maeda I. deGraw D. Kitano M. Matsushima H. Sakaji H. Izumi K.. (2020). Deep reinforcement learning in agent based financial market simulation. J. Risk Financ. Manage. 13:71. doi: 10.3390/jrfm13040071

[ref18] Nabipour M. Nayyeri P. Jabani H. Shahab S. Mosavi A. (2020). Predicting stock market trends using machine learning and deep learning algorithms via continuous and binary data; a comparative analysis. IEEE Access 8, 150199–150212. doi: 10.1109/ACCESS.2020.3015966

[ref19] Navon A. Keller Y. (2017). Financial time series prediction using deep learning. Electric Res. Archive. doi: 10.48550/arxiv.1711.04174

[ref20] Nguyen N. Trung N. Thalassinos E. Le H. (2022). The performance of shrinkage estimator for stock portfolio selection in case of high dimensionality. J. Risk Financ. Manage. 15:249. doi: 10.3390/jrfm15060249

[ref21] Nielsen L. Vassalou M. (2004). Sharpe ratios and alphas in continuous time. J. Financ. Quant. Anal. 39, 103–114. doi: 10.1017/S0022109000003902

[ref23] Peck-Ling T. Choong S. (2022). Determinants of Malaysian real estate investment trusts (m-reits) risk-adjusted performance during the period of property oversupply. J. Contemp. Issues Thought 12, 10–23. doi: 10.37134/jcit.vol12.1.2.2022, PMID: 39891254

[ref24] Pyo S. Lee J. Cha M. Jang H. (2017). Predictability of machine learning techniques to forecast the trends of market index prices: hypothesis testing for the Korean stock markets. PLoS One 12:e0188107. doi: 10.1371/journal.pone.0188107, PMID: 29136004 PMC5685607

[ref25] Razouk A. (2023). Performance evaluation of technical indicators for forecasting the Moroccan stock index using deep learning. Indonesian J. Electr. Eng. Comput. Sci. 32:1785. doi: 10.11591/ijeecs.v32.i3.pp1785-1794

[ref26] Saha V. (2023). Predicting future cryptocurrency prices using machine learning algorithms. J. Data Anal. Inf. Proc. 11, 400–419. doi: 10.4236/jdaip.2023.114021

[ref9002] Sari Y. Indrabudiman A. (2024). The role of artificial intelligence (AI) in financial risk management. Formosa Journal of Sustainable Research. 3, 2073–2082.

[ref27] Siva R. (2024). Analyzing the machine learning methods to predict bitcoin pricing. World J. Adv. Res. Rev. 21, 1288–1294. doi: 10.30574/wjarr.2024.21.1.0074

[ref28] Song D. Baek A. Kim N. (2021). Forecasting stock market indices using padding-based fourier transform denoising and time series deep learning models. IEEE Access 9, 83786–83796. doi: 10.1109/ACCESS.2021.3086537

[ref29] Sonkavde G. (2023). Forecasting stock market prices using machine learning and deep learning models: a systematic review, performance analysis and discussion of implications. Int. J. Financ. Stu. 11:94. doi: 10.3390/ijfs11030094

[ref30] Sun G. (2024). Cryptocurrency price prediction based on Xboost, Lightgbm and BNN. Appl. Comput. Eng. 49, 273–279. doi: 10.54254/2755-2721/49/20241414

[ref31] Tollo G. Andria J. Filograsso G. (2023). The predictive power of social media sentiment: evidence from cryptocurrencies and stock markets using NLP and stochastic Anns. Mathematics 11:3441. doi: 10.3390/math11163441

[ref32] Wang J. (2023). Optimization of quantitative investment strategies in the financial big data environment. Front. Bus. Econ. Manage. 12, 52–54. doi: 10.54097/fbem.v12i2.14595

[ref33] Wang Z. Qiao Y. Huang S. Liu H. (2021). Cvar prediction model of the investment portfolio based on the convolutional neural network facilitates the risk management of the financial market. J. Glob. Inf. Manag. 30, 1–16. doi: 10.4018/JGIM.295450

[ref34] Wang J. Sun T. Liu B. Cao Y. Wang D. (2018). “Financial markets prediction with deep learning,” in *17th IEEE International Conference on Machine Learning and Applications (ICMLA), Orlando, FL, USA*, 97–104.

[ref35] Zaar A. Benaya N. Moubtahij H. Bakir T. Mansouri A. Allati A. (2023). Ethereum cryptocurrency entry point and trend prediction using bitcoin correlation and multiple data combination. Int. J. Adv. Comput. Sci. Appl. 14:140506. doi: 10.14569/IJACSA.2023.0140506

[ref36] Zhang Z. (2023). “Comparison of LSTM and ARIMA in price forecasting: evidence from five indexes,” in *Proceedings of the 2023 2nd International Conference on Economics, Smart Finance and Contemporary Trade (ESFCT 2023).*

